# Randomized Comparison of Near-infrared Fluorescence Imaging Using Indocyanine Green and 99^m^ Technetium With or Without Patent Blue for the Sentinel Lymph Node Procedure in Breast Cancer Patients

**DOI:** 10.1245/s10434-012-2466-4

**Published:** 2012-07-03

**Authors:** Joost R. van der Vorst, Boudewijn E. Schaafsma, Floris P. R. Verbeek, Merlijn Hutteman, J. Sven D. Mieog, Clemens W. G. M. Lowik, Gerrit-Jan Liefers, John V. Frangioni, Cornelis J. H. van de Velde, Alexander L. Vahrmeijer

**Affiliations:** 1 Department of Surgery, Leiden University Medical Center, Leiden, The Netherlands; 2 Department of Radiology, Beth Israel Deaconess Medical Center, Boston, MA USA; 3 Department of Endocrinology, Leiden University Medical Center, Leiden, The Netherlands; 4 Division of Hematology/Oncology, Department of Medicine, Beth Israel Deaconess Medical Center, Boston, MA USA

## Abstract

**Background:**

Near-infrared (NIR) fluorescence imaging using indocyanine green (ICG) has the potential to improve sentinel lymph node (SLN) mapping of breast cancer. We performed a randomized clinical trial to assess the value of blue dyes when used in combination with NIR fluorescence. We also preliminarily examined the possibility of performing SLN mapping without radiotracers.

**Methods:**

Clinical trial subjects were 24 consecutive breast cancer patients scheduled to undergo SLN biopsy. All patients received standard of care using 99^m^ technetium-nanocolloid and received 1.6 mL of 500 μM ICG injected periareolarly. Patients were randomly assigned to undergo SLN biopsy with or without patent blue. To assess the need for radiocolloids to localize the SLN or SLNs, the surgeon did not use the handheld gamma probe during the first 15 min after the axillary skin incision.

**Results:**

SLN mapping was successful in 23 of the 24 patients. No significant difference was found in signal-to-background ratio between the groups with and without patent blue (8.3 ± 3.8 vs. 10.3 ± 5.7, respectively, *P* = 0.32). In both groups, 100 % of SLNs were radioactive and fluorescent, and in the patent blue group, only 84 % of SLNs were stained blue. In 25 % of patients, the use of the gamma probe was necessary to localize the SLN within the first 15 min.

**Conclusions:**

This study shows that there is no benefit of using patent blue for SLN mapping in breast cancer patients when using NIR fluorescence and 99^m^ technetium-nanocolloid. NIR fluorescence imaging outperformed patent blue in all patients.

Sentinel lymph node (SLN) mapping is regarded as standard of care in staging of the axilla in breast cancer patients with clinically negative axillary lymph nodes.^1^ To locate the SLN, different techniques can be used. Combining a radiotracer and blue dye staining achieves the highest identification rates (95–97 %) and is therefore preferred.^2^–^5^ However, both methods have several disadvantages. Radioactive colloids require involvement of a nuclear physician and do not provide visual information intraoperatively; further, the time window in which they can be used is short, thanks to the brief half-life of 99^m^ technetium (^99m^Tc). Moreover, a substantial portion of patients undergo SLN mapping with only a blue dye used because radioactive isotopes are not available in every medical center. The percentage of patients in whom only blue dye staining is used for SLN mapping varies from 4 % to 50 % in developed countries.^6^–^9^ Blue dyes cannot be seen through skin and fatty tissue, and they permit only limited visualization of afferent lymphatic vessels. In cases of lumpectomy, the tattoos on the breast can be seen up until several months after blue dye injection.

As a result of these disadvantages, optical imaging using the near-infrared (NIR) fluorescence lymphatic tracer indocyanine green (ICG) has been put forward as an alternative for, or an addition to, conventional SLN mapping. the feasibility of this technique has been extensively reported in breast cancer patients and other cancer types.^10^–^17^ A dose-finding study performed by our group previously demonstrated that NIR fluorescence using a dose of 500 μM ICG adsorbed to human serum albumin was most convenient.^18^ A follow-up double-blind randomized clinical trial demonstrated no advantages of premixing ICG with human serum albumin in comparison to ICG alone at a dose of 500 μM for NIR fluorescence SLN mapping.^19^

In these previous clinical trials, the NIR fluorescence signal of ICG was consistently visualized earlier than the blue dye staining. In the total 60 SLNs detected in the two studies (*n* = 42 patients), only 48 (80 %) were stained blue, while 60 (100 %) of the SLNs could be detected with NIR fluorescence.^18^,^19^ Therefore, NIR fluorescence imaging has the potential to replace blue dyes in SLN mapping in breast cancer patients. If the use of blue dye staining could be omitted, disadvantages like tattooing of the breast and blue staining of the surgical field in case of a lumpectomy could be prevented. Furthermore, the use of blue dyes may interfere with NIR fluorescence imaging by absorbing the fluorescent light and thereby decreasing the NIR fluorescent signal. Finally, anaphylactic reactions to blue dyes, although rare, can be life-threatening. Because NIR fluorescent light penetrates relatively deep into tissue (up to 0.5–1 cm), it also has the potential to replace the use of radiotracers. However, omitting radiotracers may only be reserved for selected patients—for example, those with a sufficiently low body mass index (BMI).

In the current randomized clinical trial, the added value of the use of blue dye staining in combination with NIR fluorescence and radiotracer was assessed. The concordance between NIR fluorescence, radiocolloids, and patent blue was assessed, and the possibility of performing SLN mapping without radiotracers was preliminarily explored.

## Methods

### Preparation of ICG

ICG (25-mg vials) was purchased from Pulsion Medical Systems (Munich, Germany) and was resuspended in 10 mL of sterile water for injection to yield a 2.5-mg/mL (3.2 mM) stock solution. To obtain a 500 μM dilution of ICG, 7.8 mL of the 3.2 mM ICG solution was diluted in 42.8 mL of sterile water. In a previous study, we determined that the optimal dose of ICG lies between 400 and 800 μM.^18^ Therefore, a dose of 500 μM was chosen.

### Intraoperative NIR Imaging System (Mini-FLARE)

SLN mapping was performed using the Mini-Fluorescence-Assisted Resection and Exploration (Mini-FLARE) image-guided surgery system, as described earlier.^18^ Briefly, the system consists of two wavelength-isolated light sources: a white light source, generating 26,600 lx of 400 to 650 nm light, and a NIR light source, generating 7.7 mW/cm^2^ of 760-nm light. Color video and NIR fluorescence images are simultaneously acquired and displayed in real time using custom optics and software that separate the color video and NIR fluorescence images. A pseudo-colored (lime green) merged image of the color video and NIR fluorescence images is also displayed. The imaging head is attached to a flexible gooseneck arm, which permits positioning of the imaging head at extreme angles virtually anywhere over the surgical field. For intraoperative use, the imaging head and imaging system pole stand are wrapped in a sterile shield and drape (Medical Technique, Tucson, AZ).

### Clinical Trial

This randomized, single-institution trial comparing NIR fluorescence SLN mapping with or without patent blue was approved by the medical ethics committee of the Leiden University Medical Center and was performed in accordance with the ethical standards of the Helsinki Declaration of 1975. This study was registered with the Netherlands Trial Registry (NTR2674). All patients planning to undergo a SLN procedure for invasive breast cancer or high-risk carcinoma-in-situ were eligible for participation in the trial. Patients had clinically negative axillary nodes as assessed by palpation and ultrasonography. Exclusion criteria were pregnancy, lactation, or an allergy to iodine, shellfish, or ICG. All patients provided informed consent, and data were anonymized.

As part of the SLN procedure, patients were injected periareolarly with approximately 100 MBq ^99m^Tc-nanocolloid the day before surgery. Before the start of the operation, patients were randomly assigned to receive or not receive an injection with patent blue. Patients were randomized by the Department of Surgery, and treatment allocation was performed by block randomization. For patients randomized to be injected with patent blue, 1 mL total of patent blue (Bleu Patenté V, Guerbet, Brussels, Belgium) was injected intradermally and periareolarly at four sites before the start of the operation. All patients were intradermally and periareolarly injected with 1.6 mL total of 500 μM ICG at four sites before the start of the operation. Patent blue and ICG injections were performed by the surgeon. Subsequently, gentle pumping pressure was applied to the injection site for 1 min. After surgical scrubbing and sterile covering of the operation field, NIR fluorescence imaging was performed with the imaging head of the Mini-FLARE at an approximately 30-cm distance to the surgical field. The lights in the operating room were turned off, and the surgical field was illuminated at a high fluence rate using the white light surgical luminary of the Mini-FLARE imaging system. Camera exposure times were between 5 to 250 ms. A SLN exhibiting a signal-to-background ratio (SBR) of ≥ 1.1 in situ was considered positive by NIR fluorescence.

To assess the need for radiocolloids to localize the SLN or SLNs, the surgeon did not use the handheld gamma probe during the first 15 min of the operation (starting from the axillary skin incision). In case the SLN or SLNs were not localized using only ICG or ICG in combination with patent blue within the first 15 min, the surgeon was allowed to use the handheld gamma probe for SLN localization.

Routine histopathological frozen analysis of SLNs was performed during surgery. After frozen section analysis, SLNs were fixed in formalin and embedded in paraffin for routine hematoxylin and eosin staining and immunohistopathological staining for AE1/AE3 at three levels, with an interval of 150 to 250 μm, according to the Dutch guidelines for SLN analysis. Patients underwent an axillary lymph node dissection if the SLN was found to contain metastases. If micrometastases (<0.2 mm) or isolated tumor cells were found, no axillary lymph node dissection was performed.

### Power Calculation and Statistical Analysis

To show noninferiority, a power calculation based on data from our previous studies revealed that 24 patients were needed to achieve 91 % power to detect a difference of 5.0 in SBR between the two groups, with the null hypothesis that the mean SBR of each group is 10.0 plus or minus the standard deviation of 3.5; and the alternative hypothesis that the mean of the group without blue dye is 15.0 with a significance level of 0.05 by a two-sided, two-sample *t* test.^18^,^19^ For statistical analysis, SPSS software (version 16.0; SPSS, Chicago, IL) was used. To compare patient characteristics, SBR and the number of SLNs identified between the patent blue group and the group without patent blue, the independent-sample *t* test and chi-square test were used. *P* < 0.05 was considered significant.

## Results

### Patient and Tumor Characteristics

Twenty-four consecutive breast cancer patients undergoing SLN mapping using ^99m^Tc-nanocolloid and ICG were randomized to be injected with or without patent blue. The median age of the included patients was 59 (range 39–75) years, and the median BMI was 24 (range 19–47) kg/m^2^. BMI was significantly higher in the group without patent blue (*P* = 0.042). Other patient, tumor, and treatment characteristics were equally distributed over the treatment groups (Table 1). The average time between injection of ICG and the skin incision was not significantly different between treatment groups and was 15.2 ± 3.0 min and 13.2 ± 3.0 min for the patient groups with and without patent blue, respectively. No adverse reactions associated with the use of ICG or the Mini-FLARE imaging system occurred. No postoperative complications of the SLN procedure were observed.

**Table 1:** Patient and tumor characteristics

Characteristic	Patent blue (*n* = 12)	No patent blue (*n* = 12)	*P*
Age (y), median (range)	54 (39–75)	67 (48–71)	0.15
BMI (kg/m^2^), (median, range)	23.5 (19–34)	28 (20–47)	0.042
Skin type^a^			0.62
II	2 (17 %)	2 (17 %)	
III	10 (83 %)	10 (83 %)	
Previous procedure of breast			0.54
Excision fibroadenoma	1 (8 %)	0 (0 %)	
NAC	2 (17 %)	1 (8 %)	
Neoadjuvant hormone therapy			0.31
Multifocal	0 (0 %)	1 (8 %)	0.41
Tumor side	0 (0 %)	1 (8 %)	
Left	6 (50 %)	7 (59 %)	
Right	6 (50 %)	5 (42 %)	
Tumor localization			0.69
Upper outer	7 (59 %)	7 (59 %)	
Lower outer	0 (0 %)	0 (0 %)	
Lower medial	0 (0 %)	0 (0 %)	
Upper medial	3 (25 %)	3 (25 %)	
Central	2 (17 %)	2 (17 %)	
Type of operation			0.36
Mastectomy	2 (17 %)	3 (25 %)	
Wide local excision	9 (75 %)	9 (75 %)	
SLN biopsy only	1 (8 %)	0 (0 %)	
Pathological tumor size (mm), median (range)	15 (5–35)	16 (5–50)	0.12
Histological type			1
Infiltrating ductal type adenocarcinoma	10 (83 %)	10 (83 %)	
Infiltrating lobular type adenocarcinoma	1 (8 %)	1 (8 %)	
DCIS	1 (8 %)	1 (8 %)	
Histological grade			0.56
I	4 (33 %)	3 (25 %)	
II	4 (34 %)	3 (25 %)	
III	3 (2 %)	5 (42 %)	
No grading possible (DCIS)	1 (8 %)	1 (8 %)	

*DCIS* ductal carcinoma-in-situ

^a^Skin type II indicates white: usually burns easily and tans minimally (Northern European); skin type III indicates white (average): sometimes burns and tans gradually to light brown (Central European)

### Intraoperative NIR Fluorescence Imaging

**Fig. 1:**
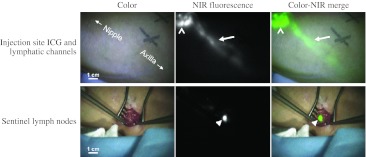
NIR fluorescence imaging during SLN mapping in a breast cancer patient. *Top row,* the periareolar injection site (*open arrowhead*) and an afferent lymphatic channel (*arrow*) are clearly visualized. *Bottom row,* identification of the SLN (*arrowhead*) with NIR fluorescence imaging is demonstrated 10 min after incision. Camera exposure times were 30 ms (*top row*) and 100 ms (*bottom row*). Scale bars = 1 cm. Patent blue was omitted in this patient

In 23 of 24 patients, at least one SLN (Fig. 1) was identified (Table 2). In 1 patient included in the group without patent blue, no SLN could be detected, even when using the gamma probe. A total of 19 and 18 SLNs were resected in groups with and without patent blue, respectively. In the patent blue group, 19 (100 %) of 19 of SLNs were NIR fluorescent, 17 (89 %) of 19 SLNs were radioactive, and 16 (84 %) of 19 SLNs were blue. In the group without patent blue, 18 (100 %) of 18 SLNs were NIR fluorescent and 18 (100 %) of 18 of SLNs were radioactive. The afferent lymphatics were visualized percutaneously in 83 % and 75 % of patients in the groups with and without patent blue, respectively. No significant difference was observed. In all patients who received patent blue, the NIR fluorescence signal in the SLN was detected before the patent blue was visualized.

**Table 2:** SLN identification results

Characteristic	Total (*n* = 24)	Patent blue (*n* = 12)	No patent blue (*n* = 12)	*P*
No. of SLNs identified	37	19	18	
No. of SLNs identified per patient				0.54
0	1 (4 %)	0 (0 %)	1 (8 %)	
1	12 (50 %)	7 (58 %)	5 (42 %)	
2	9 (38 %)	4 (33 %)	5 (42 %)	
3	1 (4 %)	1 (8 %)	0 (0 %)	
4	1 (4 %)	0 (0 %)	1 (8 %)	
No. of SLNs identified, average ± SD	1.5 ± 0.8	1.6 ± 0.7	1.5 ± 1.0	0.81
Method of detection				
Radioactive	35 (95 %)	17 (89 %)	18 (100 %)	
Blue	16 (84 %)	16 (84 %)	0 (0 %)	
Fluorescent	37 (100 %)	19 (100 %)	18 (100 %)	
SBR	9.2 ± 4.8	8.3 ± 3.8	10.3 ± 5.7	0.32
Percutaneous lymph drainage visualization				0.84
Yes	12 (50 %)	6 (50 %)	6 (50 %)	
Partial	7 (29 %)	4 (33 %)	3 (25 %)	
No	5 (21 %)	2 (17 %)	3 (25 %)	
Time between injection and skin incision (min), average ± SD	14.8 ± 3.3	15.2 ± 3.0	13.8 ± 3.9	0.17
Time between skin incision and SLN resection (min), average ± SD	12.2 ± 7.9	12.4 ± 7.7	18.1 ± 18.9	0.35
Histology of SLN				0.10
Negative	16 (67 %)	(50 %)	10 (83 %)	
Isolated tumor cells	4 (17 %)	(33 %)	0 (0 %)	
Micrometastases	2 (8 %)	0 (0 %)	2 (17 %)	
Macrometastases	2 (8 %)	2 (17 %)	0 (0 %)	
Axillary lymph node dissection				0.14
No	22 (92 %)	10 (83 %)	12 (100 %)	
Yes	2 (8 %)	2 (17 %)	0 (0 %)	

**Fig. 2:**
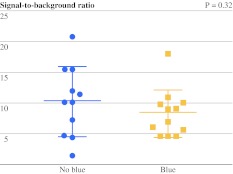
Difference in brightness of SLNs between treatment groups. SBRs (mean ± SD) of breast SLNs are plotted. The SBRs of the groups with and without patent blue were not significantly different

The average brightness of the SLN, expressed in SBR, was 8.3 ± 3.8 and 10.3 ± 5.7 for the groups with and without patent blue, respectively (Fig. 2; Table 2). No significant difference in SBR was observed between the treatment groups (*P* = 0.32). The average time between skin incision and SLN identification was 12.4 ± 7.7 min and 18.1 ± 18.9 min for the groups with and without patent blue, respectively. No significant difference was observed (*P* = 0.35). In 1 patient in the group without patent blue, the time between skin incision and SLN detection was 66.91 min. This patient received neoadjuvant chemotherapy (NAC), and the SLN was located in the area next to the latissimus dorsi muscle. Excluding this outlying data, the average time between skin incision and SLN identification was 12.4 ± 7.7 min and 13.2 ± 10.3 for the groups with and without patent blue, respectively (*P* = 0.83).

**Fig. 3:**
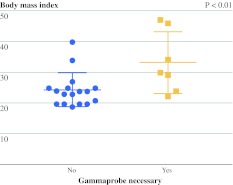
Influence of BMI on the necessity of using the gamma probe. The BMI of patients was plotted. The average BMI of patients in whom the gamma probe was needed for SLN identification (33.1 ± 9.9 kg/m^2^) was significantly higher than in patients in whom the gamma probe could be omitted (24.4 ± 5.4 kg/m^2^) (*P* < 0.01)

To assess the need for radiotracers to localize the SLNs, the surgeon did not use the handheld gamma probe during the first 15 min starting from skin incision. After the first 15 min, the use of the handheld gamma probe was necessary to locate the SLN or SLNs in 2 patients in the patent blue group and 4 patients in the group without patent blue. In all other patients, the handheld gamma probe was only used to verify the SLN for radioactivity ex vivo and to learn whether any SLNs were missed. The average BMI of patients in whom the gamma probe was needed for SLN identification (33.1 ± 9.9 kg/m^2^) was significantly higher than in patients in whom the gamma probe could be omitted (24.4 ± 5.4 kg/m^2^; *P* < 0.01; Fig. 3).

## Discussion

NIR fluorescence imaging has been extensively described as a tool for the SLN procedure in various types of cancer. In previous work, we demonstrated that a dose of 500 μM of ICG, without premixing with human serum albumin, was optimal to perform NIR fluorescence SLN mapping in breast cancer.^18^,^19^ In the current randomized clinical trial, the added value of patent blue staining, when used in combination with NIR fluorescence, was assessed in breast cancer patients undergoing the SLN procedure. Regarding the time needed to identify the SLN, no significant difference between the two treatment groups was observed. In 1 patient in the group without patent blue, the SLN mapping lasted 67 min. A possible explanation for this relatively long time to locate the SLN could be that this patient was treated with NAC. It has been proposed that NAC alters the lymphatic drainage network, thereby possibly hampering the accuracy of the SLN procedure.^20^ This could possibly be an explanation for the difficult identification of the SLN in 1 of our patients. However, in the current study, 2 more patients were treated with NAC, and in those patients, time between skin incision and SLN identification was in concordance with the average time between skin incision and the SLN identification observed in all other patients (approximately 13 min). A meta-analysis of studies that examined the SLN procedure in patients treated with NAC included 1273 patients and reported an identification rate of 90 % and a false-negative rate of 12 %.^21^ A more recent meta-analysis reported similar results.^22^ These results are not significantly different from meta-analyses of SLN biopsy in patients who are naive to chemotherapy.^23^ Nonetheless, the results of SLN biopsy after NAC remain conflicting. The prospective multicenter German SENTINA trial is currently accruing patients to evaluate the accuracy of the SLN procedure after NAC.

Comparing the patient groups with and without patent blue, the brightness of the SLNs was higher in the latter group (difference in SBR 2.0); however, this difference was not statistically significant. Furthermore, in the patent blue group, only 84 % of NIR fluorescent SLNs were stained blue, which is in concordance with previous studies.^18^,^19^ Patent blue and ICG were injected at the same time before surgery, at the same location, and by the same person. Furthermore, ICG and patent blue have comparable hydrodynamic diameters.^24^,^25^ A potential reason that SLNs may not stain blue but are NIR fluorescent is the sensitivity of NIR fluorescence imaging systems, which can detect far lower doses of tracer compared to blue dye staining.^11^,^26^,^27^ These results indicate that NIR fluorescence using ICG can replace blue dye staining. Such replacement may have several advantages. First, the use of blue dyes stains the surgical field an unnatural color that persists for several months after surgery. Second, blue dyes cannot be visualized when covered by overlying tissue, while ICG can be detected through millimeters to a centimeter of overlying tissue. Third, in many cases, ICG can be seen percutaneously; this allows lymphatic mapping before surgery, which could decrease the time to identify the SLN.

An obvious next step in the optimization of the SLN procedure would be to evaluate the need for radiotracers. Although radiotracers have superior tissue penetration, they expose caregivers and patients to ionizing radiation, and they can only be detected with a gamma probe, which does not provide the surgeon with visual information. Furthermore, the time window for SLN identification is short as a result of the short half-life (6 h) of ^99m^Tc. To explore the necessity of intraoperative radiotracers in addition to NIR fluorescence, the surgeon was not allowed to use the gamma probe during the first 15 min of the surgery. In 6 (25 %) of 24 patients, the surgeon could not identify the SLN within the first 15 min, and in 1 patient, no SLN could be detected at all. In the current study, the average BMI of patients in whom the gamma probe was used for SLN identification was significantly higher than in patients in whom the gamma probe was not necessary. These results are in concordance with previous studies that showed a significant correlation between time to identify the SLN and BMI.^28^,^29^ Furthermore, when only patent blue is used for SLN mapping, the SLN detection rate is significantly higher in patients with a BMI of <30 kg/m^2^ compared to patients with a BMI of >30 kg/m^2^.^30^ This suggests that BMI plays an important role in selecting patients eligible for NIR fluorescence SLN mapping without the use of radiotracers. However, the current study was not powered to compare the sensitivity of NIR fluorescence and radiocolloids; this ought to be addressed in a sufficiently powered clinical trial in the future. Furthermore, because larger series will be required to determine the safety and SLN identification rate when radiotracers are omitted, the use of NIR fluorescence imaging using ICG as a lymphatic tracer is at present particularly attractive to hospitals unable to work with radioactive isotopes.

Another approach within the field of SLN mapping is combining fluorescence and radioactivity in one lymphatic tracer by simply premixing ICG and ^99m^Tc-nanocolloid (complex ICG-^99m^Tc-NanoColl), which has been performed in prostate cancer patients.^17^ By using this multimodal tracer injected by the nuclear physician before surgery, time of surgery will probably be shortened because no dye injection and massage are needed in the surgical theater. A clinical trial in breast cancer patients using this hybrid multimodal radiocolloid is currently being conducted in our center.

In conclusion, this randomized trial showed no advantage of the use of patent blue for the SLN procedure in breast cancer when NIR fluorescence and radiotracers are used. When we combine these results with results from our previous work, we recommend that a dose of 500 μM ICG injected in a total of 1.6 mL be used for NIR fluorescence SLN mapping in breast cancer patients; patent blue can be omitted.
